# Construction of a kiwifruit yeast two-hybrid cDNA library to identify host targets of the *Pseudomonas syringae* pv. *actinidiae* effector AvrPto5

**DOI:** 10.1186/s13104-019-4102-x

**Published:** 2019-01-28

**Authors:** Karthikeyan Dharmaraj, Wei Cui, Erik H. A. Rikkerink, Matthew D. Templeton

**Affiliations:** 1 School of Biological Sciences, University of Auckland, Private Bag 92019, Auckland, 1142 New Zealand; 2 The New Zealand Institute for Plant and Food Research Limited, Private Bag 92169, Auckland, 1142 New Zealand; 3 Present Address: Plant Health and Environment Laboratory, Ministry for Primary Industries, 231 Morrin Road, Auckland, 1072 New Zealand

**Keywords:** Host target, Bacterial canker, Heavy metal-associated protein, cDNA library, Type three secreted effector

## Abstract

**Objective:**

Bacterial canker is a destructive disease of kiwifruit caused by the Gram-negative bacterium *Pseudomonas syringae* pv. *actinidiae* (*Psa*). To understand the disease-causing mechanism of *Psa*, a kiwifruit yeast two-hybrid cDNA library was constructed to identify putative host targets of the *Psa* Type Three Secreted Effector AvrPto5.

**Results:**

In this study, we used the Mate & Plate™ yeast two-hybrid library method for constructing a kiwifruit cDNA library from messenger RNA of young leaves. The constructed library consisted of 2.15 × 10^6^ independent clones with an average insert size of 1.52 kb. The screening of the kiwifruit yeast two-hybrid cDNA library with *Psa* AvrPto5 revealed the interaction of a V-type proton ATPase subunit-H, a proline rich-protein and heavy metal-associated isoprenylated plant protein 26. Among these, heavy metal-associated isoprenylated plant protein 26 showed a positive interaction with *Psa* AvrPto5 as both prey and bait.

**Electronic supplementary material:**

The online version of this article (10.1186/s13104-019-4102-x) contains supplementary material, which is available to authorized users.

## Introduction

Kiwifruit (*Actinidia* spp.) is one of the most valuable horticulture crops of the world, generating 2.7 billion USD in 2017 [1]. In recent years, kiwifruit cultivation faced a production constraint when *Pseudomonas syringae* pv. *actinidiae* (*Psa*), a phytopathogenic Gram-negative bacterium responsible for the bacterial canker disease in kiwifruit, caused severe losses worldwide [2]. Five *Psa* biovars (1, 2, 3, 5 and 6), have been defined based on their place of origin, variation in the accessory genome and the symptoms caused [3–5]. Strains from these five biovars can cause shoot die-back and cankers in trunks; a severe symptom that can lead to the death of the kiwifruit vine. Gram-negative plant and animal bacterial pathogens use a Type Three Secretion System to deliver effectors into the host [6, 7]. Type Three Secreted Effectors (T3SEs) are necessary for pathogen virulence in susceptible plants but, conversely, may induce an immunity-associated hypersensitive reaction in plants harbouring a resistance gene [8]. Several bacterial T3SE proteins can inhibit plant defences [9]; often, the mechanism behind this inhibition is unclear. The identification and characterization of the host target (s) of the effector may reveal these mechanisms.

The comparison of the accessory genomes of biovar 1, 2, 3, 5 and 6 has identified effector genes common to all biovars. From this subset of effectors, *Psa avrPto5* (Gene ID IYO_020425) was selected for host target identification in this study. Its homologue from the tomato bacterial speck pathogen *Pseudomonas syringae* pv. *tomato* DC3000 (*Pto*_DC3000_) *avrPto1*, has been investigated extensively in tomato and *Arabidopsis* [10]. A sequential knock-down study of 28 T3SEs in *Pto*_DC3000_ and their subsequent re-introduction into the pathogen demonstrated an increase in virulence on *N. benthamiana* leaves when *avrPto1* or *avrPtoB* was re-introduced [11]. From this, it was postulated that host target identification with *avrPto5* could reveal critical components of the *Psa* virulence mechanism in kiwifruit. To identify host targets, a Y2H system was used, owing to its tractability, the ability to observe an interaction in vivo [12], and its successful use with identification of host targets for other plant pathogen effectors [13–15]. In this study, an in vivo kiwifruit cDNA library was generated, assessed for its essential criteria, and screened using *Psa* AvrPto5 as bait.

## Main text

### Methods

#### Total RNA isolation and integrity assessment

One-month-old ‘Hort16A’ tissue-cultured plantlets were used for total RNA extraction [16]. Total RNA integrity analysis used the Agilent Bioanalyzer 2100 (Agilent Technologies, USA) as per the manufacturer’s protocol. Total RNA samples with a RNA integrity number (RIN) over 7 were utilized for mRNA isolation using a NucleoTrap^®^ mRNA kit (MACHEREY-NAGEL, Germany).

#### Y2H cDNA library construction

Purified mRNA was used for double strand (ds) cDNA synthesis as per the manufacturer’s protocol (Clontech, USA). From ds cDNA synthesis, a 7 µL aliquot was used for gel electrophoresis (0.8% agarose) to verify ds cDNA amplification. CHROMA SPIN TE-400 columns (Clontech, USA) were used for ds cDNA purification. Purified ds cDNA and pGADT7-Rec (Clontech, USA) were co-transformed into prey yeast cells (Y187; Clontech, USA) using the YeastMaker™ yeast transformation system 2 (Clontech, Cat. No. 630439). Four days after plating, transformation efficiency and the number of independent clones were calculated as per the manufacturer’s protocol (Clontech, USA). The library cell density was also determined by haemocytometer counts, and 12 prey clones were randomly selected and analysed for the presence of prey plasmids by plasmid-specific *Hin*dIII restriction enzyme digestion and agarose gel electrophoresis.

#### Testing bait auto-activation and toxicity

A PCR-based amplification of *Psa avrPto5* with *Psa* genomic DNA and gene-specific primers (see Additional file 1: Table S1) and was ligated into the pCR™8/GW/TOPO^®^ entry vector based on the manufacturer’s protocol (Invitrogen, USA). *Psa avrPto5* entry plasmid was used in a Gateway^®^ LR reaction (Invitrogen, USA) with the bait vector (pGBG2) [17] to develop a bait plasmid containing the *avrPto5* insert. Psa AvrPto5 carrying bait yeast cells (Gold Y2H; Clontech, USA) and the prey vector (pGADT7-Rec) harbouring prey yeast cells (Y187) were generated by the YeastMaker™ yeast transformation system 2. The auto-activation and toxicity of the bait were tested based on the manufacturer’s protocol (Invitrogen, USA).

#### Y2H screening and identification of positive interactors

A concentrated bait yeast culture (*Psa avrPto5*) (4 mL) and prey yeast cDNA library (1 mL) were mixed in a sterile 2 L conical flask containing 45 mL 2× YPDA liquid medium and subsequent steps were performed as per the manufacturer’s protocol (Clontech, USA). Mated yeast cells were spread on SDA/-Leu-Trp-His medium and incubated at 30 °C for 5 days. Positively interacting clones appearing on SDA/-Leu-Trp-His medium were suspended in sterile 1× TE buffer at OD_600_ 0.4 value and tested further on a higher stringency SDA/-Leu-Trp-His-Ade medium. Prey plasmids from positively interacting yeast clones were isolated, transformed into *E. coli* using a plasmid miniprep kit (Zymo Research, USA) and standard electroporation (Bio-Rad, USA) protocols, respectively. Prey plasmids re-isolated from *E. coli* were re-transformed into prey yeast cells and mating was repeated with the bait (*Psa avrPto5*) to confirm the interactions. Diploid yeast cells possessing both plasmids and controls at OD_600_ 0.4 value were plated on SDA/-Leu-Trp, SDA/-Leu-Trp-His and four plates of SDA/-Leu-Trp-His supplemented with 1 mM to 4 mM 3-AT (3-Amino triazole), a competitive inhibitor of the histidine.

#### Y2H assay

Three kiwifruit proteins identified from the above screen, heavy metal-associated isoprenylated plant protein 26 (*AcHIPP26*), V-type proton ATPase subunit H (*AcATPase*) and proline rich-plant protein (*AcPRP*), were PCR amplified from kiwifruit cDNA with gene-specific primers (see Additional file 1: Table S1) using the kiwifruit database [18–20]. The resulting PCR amplification reaction was analysed by electrophoresis (1% agarose gel), and these genes were cloned into the prey vector (pADG2) [17] as per the manufacturer’s protocol. Subsequently, these were transformed into prey yeast cells and their interaction analysed with the bait (*Psa avrPto5*) as described above. Similarly, *AcHIPP26* and *Psa avrPto5* were generated as bait and prey respectively, to verify their interaction.

#### Kiwifruit gene models

The kiwifruit gene models referred to are available from the list of FASTA gene models on the Plant and Food Research GitHub Repository (https://github.com/PlantandFoodResearch/Red5_WGS_Manual_Annotation) using the file Acc_all_models_cds.fasta.gz which contains the cDNA sequences for the coding regions.

### Results and discussion

**Fig. 1:**
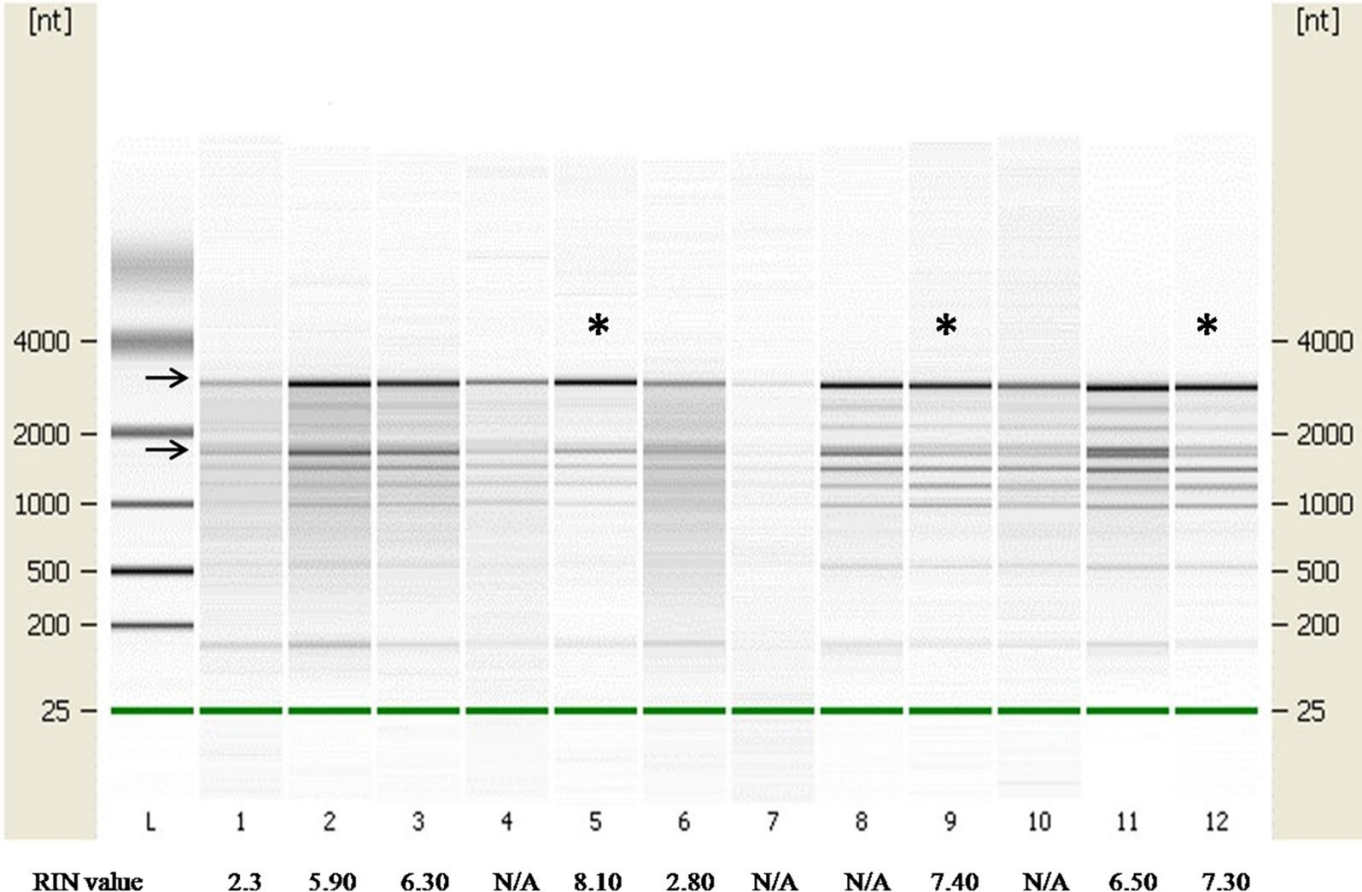
Capillary gel electrophoresis of kiwifruit total RNA samples. L-RNA ladder (*nt* nucleotide); 1–12 lanes are kiwifruit total RNA samples. *denotes total RNA samples were used for downstream processing. Arrows indicate 25S and 18S rRNA bands [32]

The integrity assessment of kiwifruit total RNA samples in the Bioanalyzer 2100 gave RIN values from 2.3 to 8.1 (Fig. 1). In contrast to animal RNA, plant RNA has characteristic 25S and 18S rRNA units, and chloroplast RNA. In this experiment, an older version of the Bioanalyzer was used which examines RNA integrity from animal tissues [21], the characteristic features of plant RNA might interfere with RIN value calculation, making RIN measurement inaccurate. This means rRNA peaks in the electropherogram should be visually examined [22]. The visual assessment of the electropherogram (see Additional file 1: Figure S1) revealed no sign of the reduction in signal magnitudes in RNA samples, indicating kiwifruit total RNA was intact. In this experiment, kiwifruit mRNA was reverse transcribed by using the SMART™ cDNA synthesis method (Clontech, USA). While verifying ds cDNA in gel electrophoresis, a cDNA smear appeared between 0.4 and 3 kb, with greatest intensity between 0.4 and 2 kb (see Additional file 1: Figure S2). This suggests kiwifruit ds cDNA with a broad range of sizes was present with suitable parameters for library generation.

The kiwifruit cDNA library was constructed by co-transforming ds cDNA and pGADT7-Rec vector into prey yeast cells. The transformation efficiency of the library was 7.1 × 10^5^ CFU/µg prey vector and the total number of independent clones was calculated as 2.15 × 10^6^ CFU/library. These findings are comparable with a study where a cDNA library generation from vernalized winter wheat in yeast using the SMART™ method showed a transformation efficiency of 5.25 × 10^5^ CFU/µg prey vector and a total number of independent clones of 2.52 × 10^6^ CFU/library [23]. This suggested optimal condition for the kiwifruit library construction. The library titre assessment by haemocytometer revealed the presence of > 1.8 × 10^7^ CFU/mL. A titre of above 1x10^7^ CFU/mL is recommended for a Y2H library (Clontech, USA). The assessment of the cDNA insert size in prey plasmids by restriction digestion demonstrated the presence of inserts in all prey plasmids (not shown). The shortest and longest cDNA insert sizes were 0.65 kb and 2.8 kb respectively, and the average cDNA insert size of the library was 1.52 kb.

A candidate Y2H assay had been performed using *Pto* AvrPto host targets (AtCERK, AtLysm, AtTIFY6B, AtTCP) [15]. Their kiwifruit orthologs were tested with *Psa* AvrPto5 in Y2H however none interacted [24]. Therefore, we chose to screen the kiwifruit cDNA library with *Psa* AvrPto5. Before initiating Y2H screening, the *Psa avrPto5* clone was investigated for toxicity and auto-activation of the histidine marker in yeast. This showed no toxicity and auto-activation of the histidine marker on SDA/-Leu-Trp-His medium (see Additional file 2: Figures S3, S4). As a result, the medium SDA/-Leu-Trp-His could be employed as a minimal medium to monitor *Psa* AvrPto5 interactions. After screening the library, 53 positive interactions were identified. The prey plasmids were first isolated from all positive yeast colonies, re-transformed into prey yeast cells and re-analyzed for the interaction characteristics. These assays revealed three prey clones having positive interactions with *Psa* AvrPto5 (see Additional file 3: Figure S5). These were identified, by sequencing the plasmid, as *Ac*HIPP26, *Ac*ATPase and *Ac*PRP (Table 1). Most of the false positives were eliminated at the first instance using SDA-Leu-Trp-His medium. The sequencing of these false positives revealed they coded for actinidin, ACC oxidase, pyruvate decarboxylase and hypothetical proteins.

**Table 1:** Positively interacting prey clones of *Psa* AvrPto5 in Y2H screening

S. no	The putative prey clones identified by sequencing	Gene model number [20]
1	Heavy metal-associated isoprenylated plant protein 26 (*Ac*HIPP26)	Acc 16317.1
2	V-type proton ATPase subunit H (*Ac*ATPase)	Acc 16945.1
3	Proline rich-plant protein (*Ac*PRP)	Acc 10774.1

**Fig. 2:**
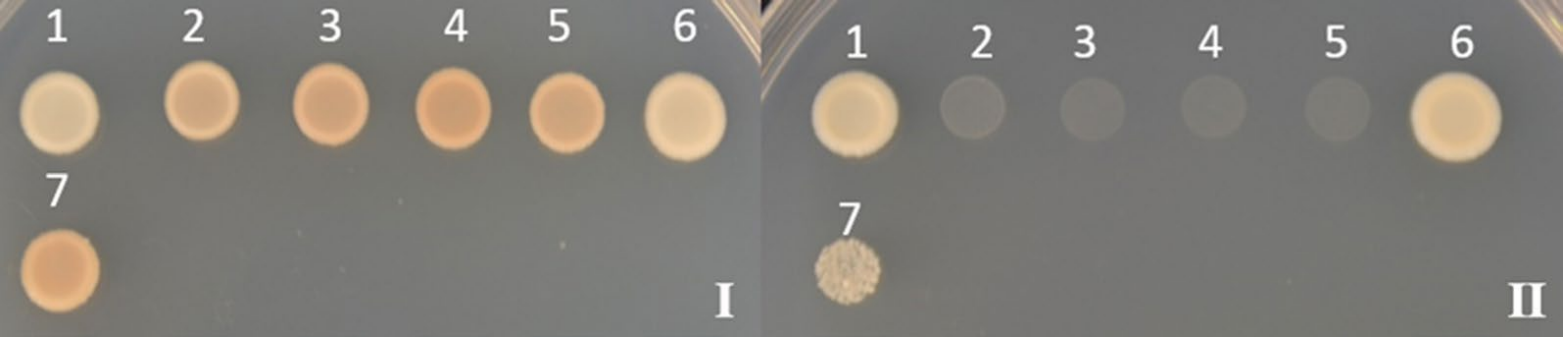
Y2H analysis of *Psa* AvrPto5 (bait) and *Ac*HIPP26 (prey). Plate I—SDA/-Leu-Trp medium; Plate II—SDA/-Leu-Trp-His + 3-AT 1 mM medium; 1—Positive interaction control (Murine p53 (bait) + SV40 large T-antigen (prey); Clontech, USA); 2—Negative interaction control (Lamin (bait) + SV40 large T-antigen (prey); Clontech, USA); 3—Negative self-activation control (Empty bait and prey vectors); 4—Bait self-activation control (*Psa* AvrPto5 (bait) + Empty prey vector); 5—Prey self-activation control (Empty bait vector + *Ac*HIPP26 (prey)); 6—True positive interaction control [33] (*Pgy* AvrB (Gene bank accession M21965) (bait) + *At*RIN4 (AT3G25070) (prey)); 7—*Psa* AvrPto5 (bait) + *Ac*HIPP26 (prey)

An reverse transcription PCR amplified full-length copy of *AcATPase*, *AcPRP* and *AcHIPP26* genes was used again to verify the interaction with the bait and *Ac*ATPase, *Ac*PRP proteins did not interact (see Additional file 3: Figure S6). The full-length copy of *Ac*HIPP26 interacted with *Psa* AvrPto5 as prey (Fig. 2) and bait (see Additional file 3: Figure S7). This indicates a true interaction between the two proteins in yeast.

The *Ac*HIPP26 is a putative metallo-chaperone protein based on alignments of its protein sequence with that of HIPP26 proteins from other plant species; it has a heavy metal-associated domain (HMA) and a C-terminus isoprenylation motif (CaaX) [25]. Several strands of evidence indicate that heavy metal-associated proteins participate in significant ways in plant-pathogen interactions [26, 27]. For example, the virulence of *Xoo* (*Xanthomonas oryzae* pv. *oryzae*) effector PthXo1 involves eliminating copper ions in rice by manipulating the Xa21-induced COPT1 and COPT5 metallo-chaperones to encourage pathogen proliferation [28]. The small HMA (sHMA) proteins are the virulence target of the *M. oryzae* effector AVR-Pik in rice [29] and the HMA domain also has a role as a host target mimic in the Pikp-1 rice resistance protein to trigger pathogen defence [30]. These examples suggest that the metallo-chaperones may have an important role in plant defence or other roles that the pathogen needs to manipulate and hence be a target for effectors. Our finding of *Ac*HIPP26 as a possible host target of *Psa* AvrPto5 adds extra weight to this hypothesis.

## Limitations

In this experiment, we identified more than fifty positive interactions with the bait. From a biological perspective, such a large number of interactions may not reflect the likely interaction *in planta*. Subsequent analyses of many of these interactions revealed them as likely false positives. We observed that partial length proteins of *Ac*ATPase and *Ac*PRP interacted strongly, however, the full length of these proteins did not interact with *Psa* AvrPto5. This discrepancy can be explained in a number of ways including false-positive interactions or an indication that domain interactions can be prevented by steric hindrance caused by the folding of other portions of the protein [31]. Sometimes such folding changes can be biologically relevant and at other times may be artefacts. Therefore, it is essential to be aware of the Y2H system disadvantages so that the technique can be employed in an effective way to accomplish its experimental aims. While the *Ac*HIPP26 protein, in particular, is a potential candidate target for *Psa* AvrPto5, the interactions identified in this analysis need to be verified by further research *in planta* to assess their biological relevance.

## Additional files

**Additional file 1: Figure S1.** Electropherogram of kiwifruit total RNA samples. The visual assessment showed the presence of prominent 25S and 18S rRNA peaks on RNA samples 2, 3, 5, 8, 9, 11 and 12.*** corresponds to three chloroplast RNA peaks [32]. Some pictures are not marked with *** because the chloroplast peaks are not prominent enough. **Figure S2.** Gel electrophoresis of kiwifruit ds cDNA synthesis. Lane L—Kb plus ladder (Invitrogen, Cat. No. 10787018); Lanes 1, 2—Kiwifruit ds cDNA; Lane 3—Mouse liver ds cDNA. **Table S1.** Gene specific primers used in this study.

**Additional file 2: Figure S3.** The bait (*Psa* AvrPto5) toxicity assay. A—Yeast cells expressing empty bait vector on SDA/-Trp medium; B—Yeast cells expressing bait (*Psa* AvrPto5) on SDA/-Trp medium. **Figure S4.** The bait (*Psa* AvrPto5) auto-activation assay. I—SDA/-Leu-Trp medium; II—SDA/-Leu-Trp-His medium. 1—Positive interaction control (Murine p53 (bait) + SV40 large T-antigen (prey); Clontech, USA); 2—Negative interaction control (Lamin (bait) + SV40 large T-antigen (prey); Clontech, USA); 3—Negative self-activation control (Empty bait and prey vectors); 4—True positive interaction control [33] (*Pgy* AvrB (bait) + *At*RIN4 (prey)); 5—*Psa* AvrPto5 (bait) and Empty prey vector.

**Additional file 3: Figure S5.** Y2H analysis of *Psa* AvrPto5 and three prey clones. Plates I—SDA/-Leu-Trp medium; Plate II—SDA/-Leu-Trp-His medium; 1—Positive interaction control (Murine p53 (bait) + SV40 large T-antigen (prey); Clontech, USA); 2—Negative interaction control (Lamin (bait) + SV40 large T-antigen (prey); Clontech, USA); 3—Negative self-activation control (Empty bait and prey vectors); 4—Bait self-activation control (*Psa* AvrPto5 (bait) + empty prey vector); 5—True positive interaction control [33] (*Pgy* AvrB (bait) + *At*RIN4 (prey)); 6—Prey self-activation control (Empty bait vector + *Ac*ATPase (prey)); 7—*Psa* AvrPto5 (bait) + *Ac*ATPase (prey); 8—Prey self-activation control (Empty bait vector + *Ac*HIPP26 (prey)); 9—*Psa* AvrPto5 (bait) + *Ac*HIPP26 (prey); 10—Prey self-activation control (Empty bait vector + *Ac*PRP (prey)); 11—*Psa* AvrPto5 (bait) + *Ac*PRP (prey). **Figure S6.** Y2H analysis of *Psa* AvrPto5 (bait) and full length *Ac*ATPase, *Ac*PRP proteins. Plate I—SDA/-Leu-Trp medium; Plate II—SDA/-Leu-Trp-His medium. 1—Positive interaction control (Murine p53 (bait) + SV40 large T-antigen (prey); Clontech, USA); 2—Negative interaction control (Lamin (bait) + SV40 large T-antigen (prey); Clontech, USA); 3—Negative self-activation control (Empty bait and prey vectors); 4—Bait self-activation control (*Psa* AvrPto5 (bait) + Empty prey vector); 5—True positive interaction control [33] (*Pgy* AvrB (bait) + *At*RIN4 (prey)); 6—Prey self-activation control (Empty bait vector + *Ac*ATPase (prey)); 7—*Psa* AvrPto5 (bait) + *Ac*ATPase (prey); 8—Prey self-activation control (Empty bait vector + *Ac*PRP (prey)); 9—*Psa* AvrPto5 (bait) + *Ac*PRP (prey). **Figure S7.** Y2H analysis of *Ac*HIPP26 (bait) and *Psa* AvrPto5 (prey). Plate I—SDA/-Leu-Trp medium; Plate II—SDA/-Leu-Trp-His + 3-AT 4 mM medium; 1—Positive interaction control (Murine p53 (bait) + SV40 large T-antigen (prey); Clontech, USA); 2—Negative interaction control (Lamin (bait) + SV40 large T-antigen (prey); Clontech, USA); 3—Negative self-activation control (Empty bait and prey vectors); 4—True positive interaction control [33] (*Pgy* AvrB (bait) + *At*RIN4 (prey)); 5—Bait self-activation control (*Ac*HIPP26 (bait) + empty prey vector); 6—Prey self-activation control (Empty bait vector + *Psa* AvrPto5 (prey)); 7—*Ac*HIPP26 (bait) +* Psa* AvrPto5 (prey).

## Abbreviations

3-AT: 3-amino triazole
Ac: *Actinidia chinensis* ‘Hort16A’
At: *Arabidopsis thaliana* (ecotype Columbia 0)
CFU: colony forming units
HMA: heavy metal-associated domain
mRNA: messenger RNA
PCR: polymerase chain reaction
*Pgy*: *Pseudomonas syringae* pv. *glycinea* race 4
*Psa*: *Pseudomonas syringae* pv. *actinidiae*
*Pto*_DC3000_: *Pseudomonas syringae* pv. *tomato* DC3000
RIN: RNA integrity number
rRNA: ribosomal RNA
SDA: synthetic dropout agar
T3SE: type three secreted effector
