# Supplementary material for: Construction of a kiwifruit yeast two-hybrid cDNA library to identify host targets of the Pseudomonas syringae pv. actinidiae effector AvrPto5
Source: BMC Res Notes. 2019 Jan 28;12:63. doi: 10.1186/s13104-019-4102-x (PMC6350409; doi:10.1186/s13104-019-4102-x)
Supplement: Supplementary file 1 — Additional file 1: Figure S1. Electropherogram of kiwifruit total RNA samples. The visual assessment showed the presence of prominent 25S and 18S rRNA peaks on RNA samples 2, 3, 5, 8, 9, 11 and 12.*** corresponds to three chloroplast RNA peaks [32]. Some pictures are not marked with *** because the chloroplast peaks are not prominent enough. Figure S2. Gel electrophoresis of kiwifruit ds cDNA synthesis. Lane L—Kb plus ladder (Invitrogen, Cat. No. 10787018); Lanes 1, 2—Kiwifruit ds cDNA; Lane 3—Mouse liver ds cDNA. Table S1. Gene specific primers used in this study. [file 13104_2019_4102_MOESM1_ESM.docx]

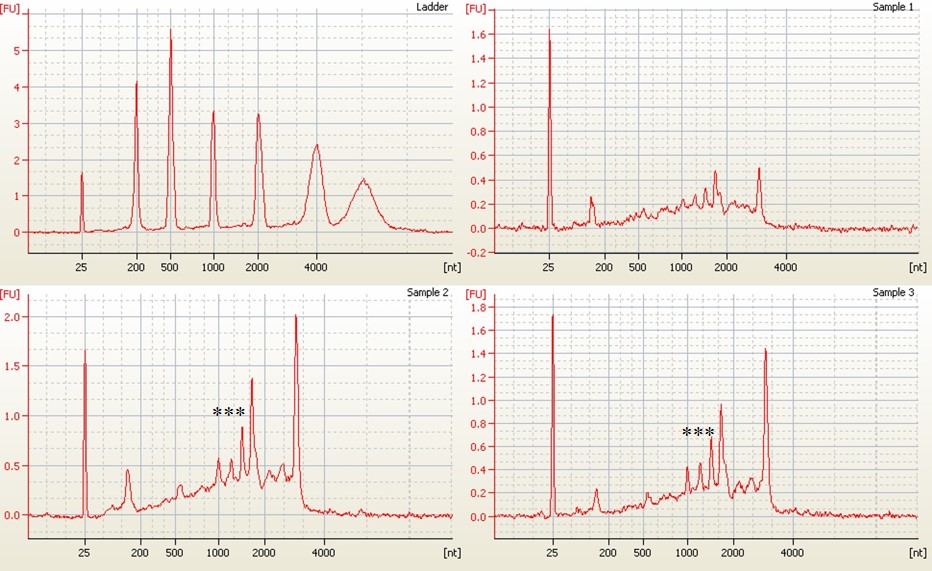


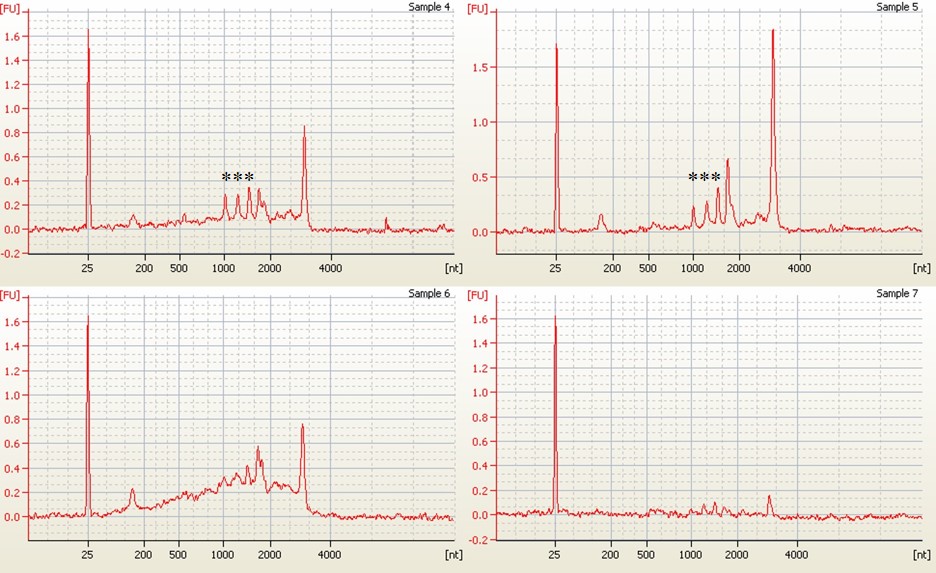


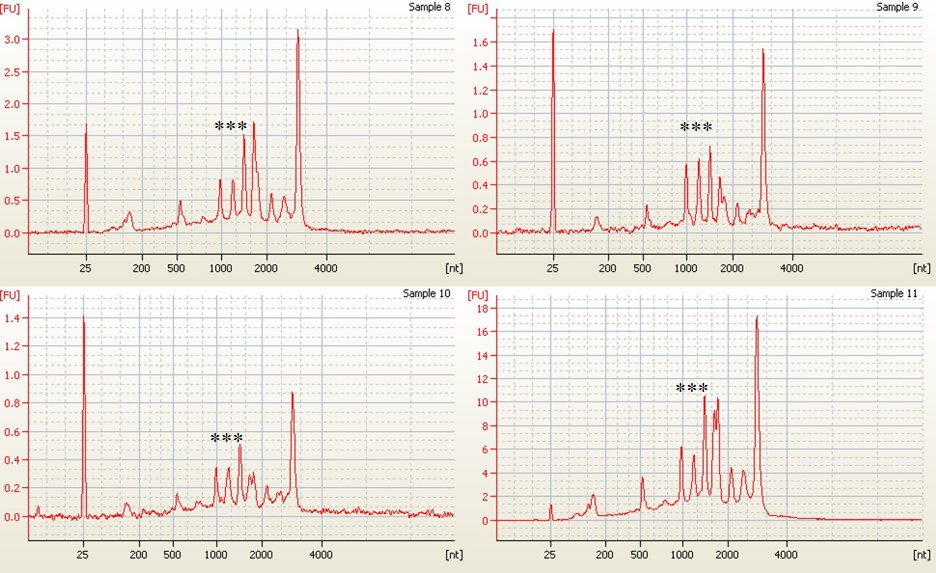


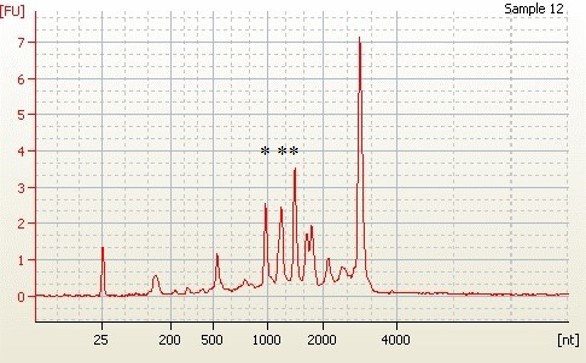


Figure S1 Electropherogram of kiwifruit total RNA samples. The visual assessment showed the presence of prominent 25S and 18S rRNA peaks on RNA samples 2, 3, 5, 8, 9, 11 and 12.*** corresponds to three chloroplast RNA peaks [32]. Some pictures are not marked with *** because the chloroplast peaks are not prominent enough.


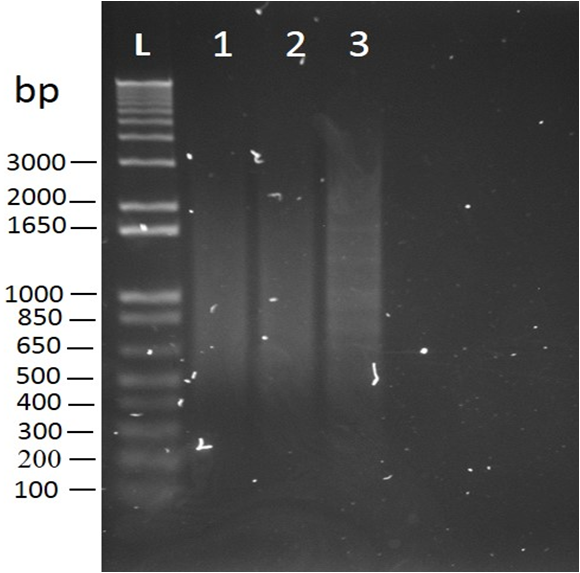


Figure S2 Gel electrophoresis of kiwifruit ds cDNA synthesis. Lane L - Kb plus ladder (Invitrogen, Cat. No. 10787018); Lanes 1, 2 - Kiwifruit ds cDNA; Lane 3 - Mouse liver ds cDNA.

Table S1 Gene specific primers used in this study

| **S.no** | **Primers name** | **Nucleotide sequence** |
| --- | --- | --- |
| 1 | *AcHIPP26* forward primer | **ATG**GGGGCTTTGGATCATCT |
| 2 | *AcHIPP26* reverse primer | **TCA**CATGACAACACAAGCGG |
| 3 | *AcATPase* forward primer | **ATG**CCGACGGAACATGCTGA |
| 4 | *AcATPase* reverse primer | **TTA**AGCCTGCAAAAAGCTCG |
| 5 | *AcPRP* forward primer | **ATG**GGGATTAATTCCCTTTG |
| 6 | *AcPRP* reverse primer | **CTA**AGGATGATGGTAAGGAG |
| 7 | *Psa* *avrPto5* forward primer | **ATG**GGAAACGTATGCGTTGG |
| 8 | *Psa* *avrPto5* reverse primer | **CTA**GCCGTTGCGGGGAGCGA |

Bold letters are the start and stop codons of the primer
