# Supplementary material for: Construction of a kiwifruit yeast two-hybrid cDNA library to identify host targets of the Pseudomonas syringae pv. actinidiae effector AvrPto5
Source: BMC Res Notes. 2019 Jan 28;12:63. doi: 10.1186/s13104-019-4102-x (PMC6350409; doi:10.1186/s13104-019-4102-x)
Supplement: Supplementary file 2 — Additional file 2: Figure S3. The bait (Psa AvrPto5) toxicity assay. A—Yeast cells expressing empty bait vector on SDA/-Trp medium; B—Yeast cells expressing bait (Psa AvrPto5) on SDA/-Trp medium. Figure S4. The bait (Psa AvrPto5) auto-activation assay. I—SDA/-Leu-Trp medium; II—SDA/-Leu-Trp-His medium. 1—Positive interaction control (Murine p53 (bait) + SV40 large T-antigen (prey); Clontech, USA); 2—Negative interaction control (Lamin (bait) + SV40 large T-antigen (prey); Clontech, USA); 3—Negative self-activation control (Empty bait and prey vectors); 4—True positive interaction control [33] (Pgy AvrB (bait) + AtRIN4 (prey)); 5—Psa AvrPto5 (bait) and Empty prey vector. [file 13104_2019_4102_MOESM2_ESM.docx]

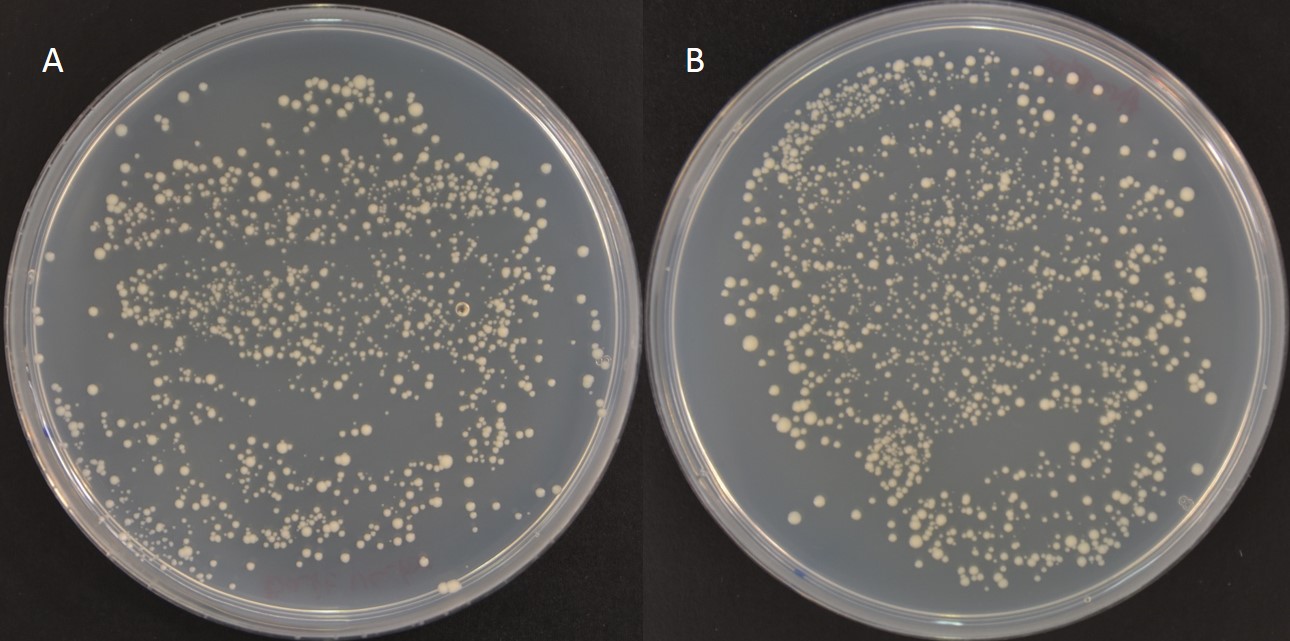


Figure S3 The bait (*Psa* AvrPto5) toxicity assay. A - Yeast cells expressing empty bait vector on SDA/-Trp medium; B - Yeast cells expressing bait (*Psa* AvrPto5) on SDA/-Trp medium.


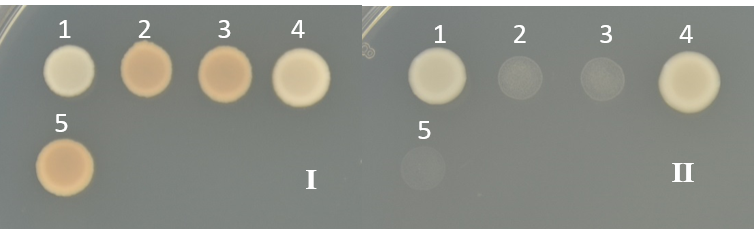


Figure S4 The bait (*Psa* AvrPto5) auto-activation assay*.* I - SDA/-Leu-Trp medium; II - SDA/-Leu-Trp-His medium. 1 - Positive interaction control (Murine p53 (bait) + SV40 large T-antigen (prey); Clontech, USA); 2 - Negative interaction control (Lamin (bait) + SV40 large T-antigen (prey); Clontech, USA); 3 - Negative self-activation control (Empty bait and prey vectors); 4 - True positive interaction control [33] (*Pgy* AvrB (bait) + *At*RIN4 (prey)); 5 - *Psa* AvrPto5 (bait) and Empty prey vector.
