# Supplementary material for: Construction of a kiwifruit yeast two-hybrid cDNA library to identify host targets of the Pseudomonas syringae pv. actinidiae effector AvrPto5
Source: BMC Res Notes. 2019 Jan 28;12:63. doi: 10.1186/s13104-019-4102-x (PMC6350409; doi:10.1186/s13104-019-4102-x)
Supplement: Supplementary file 3 — Additional file 3: Figure S5. Y2H analysis of Psa AvrPto5 and three prey clones. Plates I—SDA/-Leu-Trp medium; Plate II—SDA/-Leu-Trp-His medium; 1—Positive interaction control (Murine p53 (bait) + SV40 large T-antigen (prey); Clontech, USA); 2—Negative interaction control (Lamin (bait) + SV40 large T-antigen (prey); Clontech, USA); 3—Negative self-activation control (Empty bait and prey vectors); 4—Bait self-activation control (Psa AvrPto5 (bait) + empty prey vector); 5—True positive interaction control [33] (Pgy AvrB (bait) + AtRIN4 (prey)); 6—Prey self-activation control (Empty bait vector + AcATPase (prey)); 7—Psa AvrPto5 (bait) + AcATPase (prey); 8—Prey self-activation control (Empty bait vector + AcHIPP26 (prey)); 9—Psa AvrPto5 (bait) + AcHIPP26 (prey); 10—Prey self-activation control (Empty bait vector + AcPRP (prey)); 11—Psa AvrPto5 (bait) + AcPRP (prey). Figure S6. Y2H analysis of Psa AvrPto5 (bait) and full length AcATPase, AcPRP proteins. Plate I—SDA/-Leu-Trp medium; Plate II—SDA/-Leu-Trp-His medium. 1—Positive interaction control (Murine p53 (bait) + SV40 large T-antigen (prey); Clontech, USA); 2—Negative interaction control (Lamin (bait) + SV40 large T-antigen (prey); Clontech, USA); 3—Negative self-activation control (Empty bait and prey vectors); 4—Bait self-activation control (Psa AvrPto5 (bait) + Empty prey vector); 5—True positive interaction control [33] (Pgy AvrB (bait) + AtRIN4 (prey)); 6—Prey self-activation control (Empty bait vector + AcATPase (prey)); 7—Psa AvrPto5 (bait) + AcATPase (prey); 8—Prey self-activation control (Empty bait vector + AcPRP (prey)); 9—Psa AvrPto5 (bait) + AcPRP (prey). Figure S7. Y2H analysis of AcHIPP26 (bait) and Psa AvrPto5 (prey). Plate I—SDA/-Leu-Trp medium; Plate II—SDA/-Leu-Trp-His + 3-AT 4 mM medium; 1—Positive interaction control (Murine p53 (bait) + SV40 large T-antigen (prey); Clontech, USA); 2—Negative interaction control (Lamin (bait) + SV40 large T-antigen (prey); Clontech, USA); 3—Negative self-activatio [file 13104_2019_4102_MOESM3_ESM.docx]

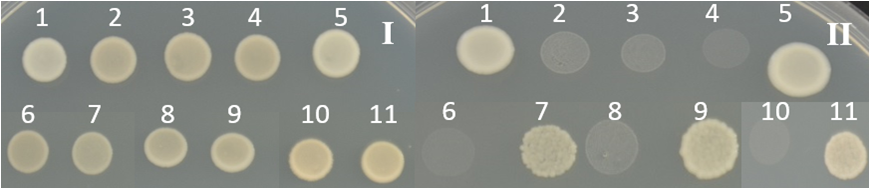


Figure S5 Y2H analysis of *Psa* AvrPto5 and three prey clones*.* Plates I - SDA/-Leu-Trp medium; Plate II - SDA/-Leu-Trp-His medium; 1 - Positive interaction control (Murine p53 (bait) + SV40 large T-antigen (prey); Clontech, USA); 2 - Negative interaction control (Lamin (bait) + SV40 large T-antigen (prey); Clontech, USA); 3 - Negative self-activation control (Empty bait and prey vectors); 4 - Bait self-activation control (*Psa* AvrPto5 (bait) + empty prey vector); 5 - True positive interaction control [33] (*Pgy* AvrB (bait) + *At*RIN4 (prey)); 6 - Prey self-activation control (Empty bait vector + *Ac*ATPase (prey)); 7 - *Psa* AvrPto5 (bait) + *Ac*ATPase (prey); 8 - Prey self-activation control (Empty bait vector + *Ac*HIPP26 (prey)); 9 - *Psa* AvrPto5 (bait) + *Ac*HIPP26 (prey); 10 - Prey self-activation control (Empty bait vector + *Ac*PRP (prey)); 11 - *Psa* AvrPto5 (bait) + *Ac*PRP (prey).


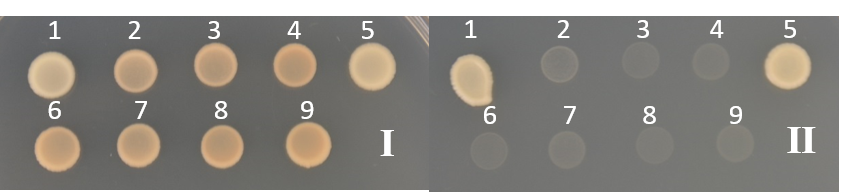


Figure S6 Y2H analysis of *Psa* AvrPto5 (bait) and full length *Ac*ATPase, *Ac*PRP proteins. Plate I - SDA/-Leu-Trp medium; Plate II - SDA/-Leu-Trp-His medium. 1 - Positive interaction control (Murine p53 (bait) + SV40 large T-antigen (prey); Clontech, USA); 2 - Negative interaction control (Lamin (bait) + SV40 large T-antigen (prey); Clontech, USA); 3 - Negative self-activation control (Empty bait and prey vectors); 4 - Bait self-activation control (*Psa* AvrPto5 (bait) + Empty prey vector); 5 - True positive interaction control [33] (*Pgy* AvrB (bait) + *At*RIN4 (prey)); 6 - Prey self-activation control (Empty bait vector + *Ac*ATPase (prey)); 7 - *Psa* AvrPto5 (bait) *+* *Ac*ATPase (prey); 8 *-* Prey self-activation control (Empty bait vector + *Ac*PRP (prey)); 9 - *Psa* AvrPto5 (bait) *+* *Ac*PRP (prey).


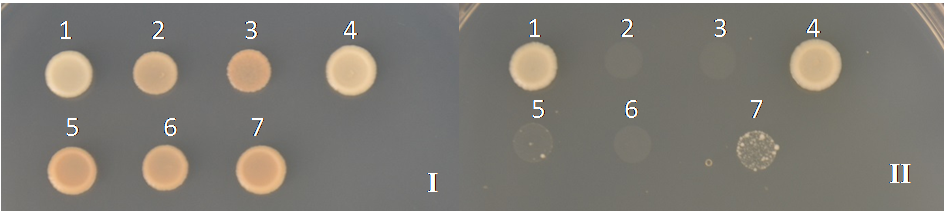


Figure S7 Y2H analysis of *Ac*HIPP26 (bait) and *Psa* AvrPto5 (prey). Plate I - SDA/-Leu-Trp medium; Plate II - SDA/-Leu-Trp-His + 3-AT 4 mM medium; 1 - Positive interaction control (Murine p53 (bait) + SV40 large T-antigen (prey); Clontech, USA); 2 - Negative interaction control (Lamin (bait) + SV40 large T-antigen (prey); Clontech, USA); 3 - Negative self-activation control (Empty bait and prey vectors); 4 - True positive interaction control [33] (*Pgy* AvrB (bait) + *At*RIN4 (prey)); 5 - Bait self-activation control (*Ac*HIPP26 (bait) + empty prey vector); 6 - Prey self-activation control (Empty bait vector + *Psa* AvrPto5 (prey)); 7 - *Ac*HIPP26 (bait) *+ Psa* AvrPto5 (prey).
